# Bioinformatics Analysis and Spatiotemporal Distribution of the fliC Gene and Its Protein Isolated from Escherichia coli-Infected Patients in Eastern Algeria

**DOI:** 10.21315/mjms2024.31.5.12

**Published:** 2024-10-08

**Authors:** Abdenassar Harrar, Rukman Awang Hamat, Mohamed Abdelhafid Hamidechi

**Affiliations:** 1 Department of Microbiology, Faculty of Nature and Life Sciences, Frère Mentouri Constantine 1 University, Ain El-Bey, Algeria; 2 Department of Microbiology and Biochemistry, Faculty of Sciences, Laboratory of Biologie: Application en Santé et Environnement, University Mohamed Boudiaf of M’sila, Algeria; 3 Department of Medical Microbiology, Faculty of Medicine and Health Sciences, Universiti Putra Malaysia, Selangor, Malaysia; 4 Department of Applied Biology, Faculty of Nature and Life Sciences, Laboratory of Microbiology Engineering and Applications, Frère Mentouri Constantine 1 University, Ain El-Bey, Algeria

**Keywords:** Escherichia coli, fliC, mutation, multifactorial statistical analysis

## Abstract

**Background:**

The *fliC* locus in *Escherichia coli* primarily encodes flagellar (H) antigens. Exploring *fliC* sequence diversity will shed light on the mechanisms of bacterial pathogenicity. This study examined the presence of *fliC* mutant strains of *E. coli* in infected patients from different age groups, sexes and sample types in eastern Algerian provinces over a span of 2 years.

**Methods:**

This retrospective, cross-sectional study involved three provinces in eastern Algeria: i) Bordj Bou Arreridj, ii) Setif and iii) Batna. A total of 75 *E. coli* isolates were obtained from the University State Hospital Centre. Two types of analyses were conducted: i) a bioinformatics analysis of the protein sequences translated from the *fliC* genes, specifically the *fliC* flagellar sequences and ii) a multifactorial statistical analysis (multiple correspondence analysis [MCA]) of the population of infected patients, considering various parameters. The *fliC* protein sequences were aligned using the Multiple Alignment using Fast Fourier Transform (MAFFT) programme. The alignment results were then visualised using the MView programme. Finally, a phylogenetic tree was constructed using the maximum likelihood algorithm in MEGA 11 software.

**Results:**

Bioinformatics analysis highlighted the strong conservation of the structures of the *fliC* protein sequences, especially at the two N- and C-terminal ends, and strong variability in the central zone. This remarkable *fliC* intersequence similarity is corroborated by the presence of protein motifs identified in the PROSITE protein motif database.

**Conclusion:**

*fliC* mutations in *E. coli* were not detected in the clinical samples of patients from hospitals in the three Algerian Provinces. Our analysis revealed that all the samples exhibited characteristics of wild-type virulent bacteria without mutations. A multicentre study is warranted for epidemiological surveillance of *fliC* mutant strains for future preventive measures.

## Introduction

The flagellum is an organelle that plays a crucial role in facilitating movement in a wide range of bacterial species. The *fliC* gene encodes a protein known as flagellin, which is the primary structural element of the filament. According to genetic studies, the N- and C-terminal domains of flagellin play crucial roles in the process of filament production. The filament core exhibits a consistent structure among bacterial species, while the outside domains display significant variation and may be entirely absent in certain instances. The observed variations can be attributed to functional adaptation, evolutionary divergence, gene regulation and gene acquisition or loss (1). Indeed, several bacterial species have demonstrated that the N-terminal and C-terminal regions do not participate in protein activity, namely, motility. Additionally, different alignment analyses have identified numerous amino acid variations within these two regions. Nevertheless, the core region of the bacteria exhibits a relatively high degree of conservation and plays a prominent role in the pathogenicity of the microorganism (2). Bacterial flagellin genes and proteins have substantial intraspecies variability, rendering them suitable for species or strain identification purposes. Nevertheless, the examination of phylogenetic data pertaining to housekeeping genes associated with virulence indicated that ancestral lineages of *Escherichia coli* acquired virulence factors throughout their molecular evolutionary process. This finding lends support to the hypothesis that the manifestation of heightened clonal virulence is a recent and derived state that emerged because of the acquisition of virulence genes, specifically the *fliC* gene, rather than being an inherent characteristic of primitive *E. coli*.

The occurrence of parallel evolution suggests that natural selection has facilitated the systematic acquisition of genes and the gradual advancement of molecular processes that enhance virulence (3). Therefore, the objective of this work was to investigate the occurrence of mutations in the amino acid residues of flagellin protein sequences from *E. coli* strains obtained from three distinct provinces in Eastern Algeria (geophylogeny) over a period of three consecutive years using bioinformatics analysis. Furthermore, an investigation was conducted to create a database for epidemiological monitoring purposes by examining the spatiotemporal distribution of this pathogen. The flagellin sequence of *E. coli* was analysed as a fundamental component for diagnostic identification.

## Methods

### Sampling Sites and Biomolecular Analyses

The pathogenic *E. coli* isolates were collected from three separate locations in Eastern Algeria: i) the Centre Hospitalo-Universitaire Saadna Mohamed Abdenour in Setif Province, which has a total of 502 beds and is located at coordinates 36°11′28″N and 5°24′11″E; ii) the University Hospital: Benflis Al-Tohamy in Batna Province, which has 540 beds and is situated at coordinates 35°31′54″N and 6°11″E and iii) the Bouzidi Lakhdar Hospital in Bordj Bou Arreridj Province, which has 240 beds and is located at coordinates 36°3′44″N and 4°46″E. Non-repetitive *E. coli* isolates were randomly collected over a period of 24 months, specifically from October 2014 to August 2016. The biomolecular analysis, which included polymerase chain reaction (PCR) and sequencing, was conducted in collaboration with the Laboratory of Medical Microbiology at the Faculty of Medical and Health Sciences, Universiti Putra Malaysia.

### Description of the Clinical Samples

A total of 75 isolates were obtained from three collection sites: i) Setif, ii) Batna and iii) Bordj Bou Arreridj Provinces. These three collection sites were selected in a non-systematic manner, without any specific statistical or spatiotemporal criteria. Samples were randomly collected from all patients who presented with *E. coli* infections during the study period. The microbiology laboratories received clinical samples from different departments within the hospital, as well as samples from outpatients. The data for this study were collected during multiple sampling periods, chosen based on our observation of a significant volume of clinical samples: October 2014–December 2014 (3 months), January 2015–February 2015 (2 months) and March 2016–August 2016 (6 months).

### Phenotypic Identification of Isolates

The isolates were identified according to basic biochemical tests using API 20E systems (Biomerieux, France). The data were interpreted according to the manufacturer’s instructions.

### Molecular Identification

#### Extraction of Bacterial DNA

For each isolate, DNA extraction was performed from one colony in a final volume of 200 μL of distilled water by incubation at 95 °C for 10 min (boiling method). Bacterial DNA was recovered after removal of cell debris (pellet) by centrifugation at 12,298 × g for 10 min. The DNA was then stored at −20 °C until further use (4, 5). DNA purity and quantity were determined prior to PCR to verify the proper concentration using a NanoDrop spectrophotometer (NanoDrop^®^ ND-1000 UV-Vis Spectrophotometer, Thermo Fisher Scientific, USA) based on absorbance readings at 260 nm and 280 nm. The DNA concentration was determined by measuring the absorbance of 1 μL at 260 nm (A260), which is equivalent to 50 ng of DNA. DNA purity was estimated by calculating the ratio of the optical density at 260 nm divided by that at 280 nm (A260/A280). An A260/A280 ratio greater than or equal to 1.7 ng/μL is expected for pure double-stranded DNA (6).

#### PCR and Gel Electrophoresis

PCR was performed using a Bio-Rad MyCycler Thermal Cycler (USA) to amplify the 16S rRNA and *fliC* genes following the protocol established by Yang et al. (7). The PCR mixture contained 12.5 μL of 2× PCR Master Mix (FastStart Taq DNA Polymerase), 1 μL of each primer, 5.5 μL of nuclease-free H_2_O and 5 μL of target DNA. The PCR steps included initial denaturation at 96 °C for 4 min, followed by denaturation at 94 °C for 1 min, primer hybridisation at 65 °C for 1 min and initial elongation at 72 °C for 1 min. A final elongation step was performed at 72 °C for 1 min.

The PCR products were subjected to electrophoresis on a 1% agarose gel at 70 V, 400 mA for 40 min. The gel was prepared with 1 g of agarose in 100 mL of 1× Tris-borate-EDTA (TBE) buffer supplemented with 0.5 μL of health view nucleic acid stain. The gel was polymerised and then immersed in a migration vessel with 1× TBE buffer. PCR products, along with a 1 kb DNA Ladder marker, were loaded with Blue Juice Loading Buffer. Gel reading was performed under a UV transilluminator and PCR-positive products were subsequently sequenced (8). Table 1 shows the primers used for PCR in the study.

#### Sequencing of the fliC Gene

PCR products were sequenced at MyTACG Bioscience Enterprise, Kuala Lumpur, Malaysia. The resulting nucleotide subsequences were assembled (forward and reverse) using CodonCode Aligner software and analysed using the BLAST programme to verify their identity against the NCBI portal GenBank sequences available at the following URL: https://www.ncbi.nlm.nih.gov/. The 75 sequences of the *fliC* genes were submitted to the GenBank database.

#### Bioinformatics Analysis

##### Multiple alignment

The *fliC* nucleic acid sequences were pre-processed before multiple sequence alignment to homogenise our data. Thus, sequences with high identity (or low dissimilarity) were removed to avoid sequence redundancy and to reduce computational time during phylogenetic construction. The pairs of sequences with low distances found on the distance matrix were constructed via the Emboss site (https://www.bioinformatics.nl/cgi-bin/emboss/distmat); therefore, those with very high identity were deleted (Supplementary Table 1). The distance scores are expressed in terms of the number of substitutions per 100 bp, which are also called dissimilarity coefficients.

Multiple sequence alignment of the *fliC* gene was performed with the Multiple Alignment using Fast Fourier Transform (MAFFT) programme (EMBL-EBI) and the results were visualised with the MView programme (EMBL, EBI). Multiple alignment of *fliC* protein sequences was performed using the ClustalW2 computer programme (EMBL 2022 | EMBL-EBI) and sequences larger than 400 amino acid residues were used to derive the maximum amount of information from the readout of this alignment. The similarity of the protein sequences of the *fliC* gene was assessed by a motif search using the PROSITE database. To do this, only sequences with sizes greater than or equal to 300 residues were retained.

##### Phylogeny of the fliC gene

Our sequences were compared to GenBank sequences using the BlastN programme (specific for noncoding nucleic sequences) for the 16S rDNA gene and the BlastX programme for the *fliC* gene. The phylogenetic analysis was performed using MEGA 11 software. The analysis was performed using multiple alignments with the MUSCLE algorithm. Subsequently, manual correction was performed to remove regions with a high frequency of gaps, specifically the 5′-phosphate and 3′-OH regions of this alignment. These regions are not favourable for phylogenetic construction because they are less informative sites and are considered divergent regions. This multiple alignment will make it possible to highlight areas in the 75 sequences whether these sequences are highly similar and/or have low similarity. The latter can be explained by very high mutation rates (substitutions or insertions/deletions) between the aligned sequences. In contrast, the areas of high similarity found in the multiple alignment indicate a high conservation of nucleotides within these sequences.

The multiple alignment results were subsequently used to construct a phylogenetic tree. Phylograms were inferred using the maximum likelihood (ML) method, with *Salmonella enterica Typhimurium* serving as the outgroup. The distance matrix was adjusted using the k2P evolutionary model. The bootstrap values were obtained from 1,000 replications (11–13).

#### Statistical Analysis

Multiple correspondence analyses (MCAs) were used to analyse the spatiotemporal distribution of the pathogens and their relationships with the analysed parameters. This study also helped to elucidate the epidemiological and pathological interrelationships within the population. The analyses were conducted using IBM SPSS version 26.0 software on Windows (IBM Corp., 2019) (14).

## Results

### General Characteristics

Information on patients with *E. coli* infections and their geographical locations were retrieved from the medical records, as shown in Table 2.

Clinical samples were obtained from 75 patients aged 1 year old–88 years old (median: 38 ± 23.21 years old). The patients infected with *E. coli* were categorised according to age group as follows: ≤ 14 years old (17 patients), 15 years old–29 years old (16 patients), 30 years old–59 years old (25 patients) and ≥ 60 years old (17 patients). Fifty-two and 23 of the patients were females and males, respectively. The clinical samples were collected from patients living in different provinces: Setif (54 patients), Batna (13 patients) and Bordj Bou Arreridj (8 patients). The data can be found in the Clinical Information Sheet (Supplementary Table 2).

### fliC Gene Sequencing

In the present study, the sequences varied in size, ranging from 924 bp to 1,864 bp, with a difference of 940 bp. On average, the size was 1,477 bp, with a standard deviation of 266 bp. The sequences were submitted to the GenBank database and their accession numbers are listed in Supplementary Table 3. The BLAST programme was used to determine functional and evolutionary relationships between sequences. BLAST results provide information on the percentage coverage, which indicates the size ratio between our sample sequences and GenBank. They also include the percentage identity, which measures the number of identical amino acid residues between two sequences. Additionally, the E-value expresses the probability of finding a similar sequence by chance in the database. As the E-value approaches zero, the error of having a sequence that is significantly different from our sample sequences becomes minimal or even zero.

### Bioinformatics Analysis

#### Multiple Alignment

**Figure 1:**
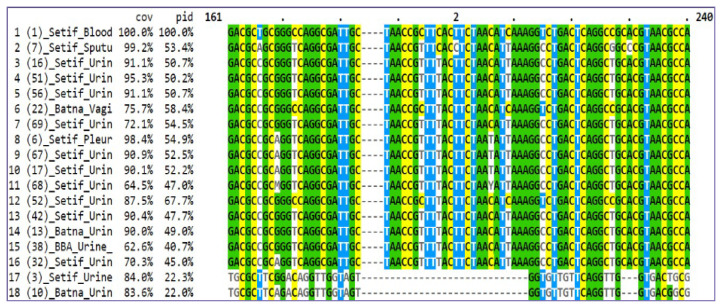
A portion of the multiple alignment of the 5′ region of the *fliC* gene sequence

Treatment of sequences to eliminate redundancy of the *fliC* gene resulted in the removal of 19 out of 75 sequences, representing 25% redundancy. The distance scores for these sequences ranged from 0.00 to 6.22. The remaining sequences (56 sequences) with dissimilarity coefficients greater than 80% were analysed (Supplementary Table 4). Multiple sequence alignment revealed similarity in the 5′ region of the sequences, indicating conservation of the *fliC* gene (Figure 1).

**Figure 2:**
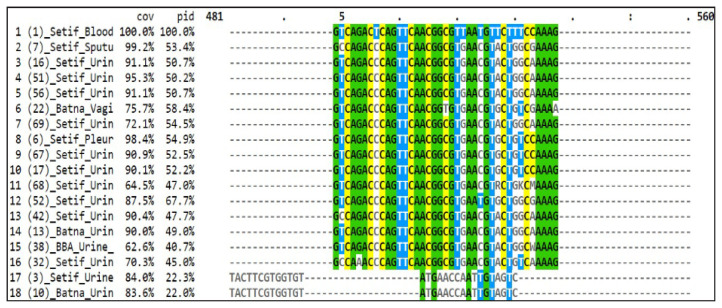
A portion of the multiple alignment of the core area of the *fliC* gene sequences

A strong dissimilarity was observed in the middle region of the multiple sequence alignment (Figure 2), indicating that the *fliC* gene is not conserved.

**Figure 3:**
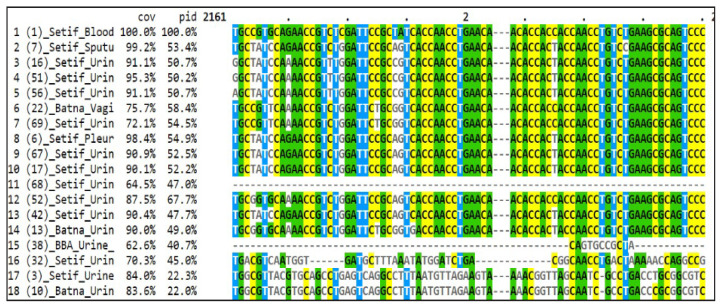
A portion of the multiple alignment of the 3′ region of the *fliC* gene sequence

The 3′ region of the multiple alignment, as shown in Figure 3, exhibited greater conservation than did the central region. It also displayed a significant amount of positional identity among sequence residues.

Multiple sequence alignment revealed that the N-terminal and C-terminal regions were highly conserved, while the core regions were highly variable (Supplementary Figures 1a, b and c).

#### Search for Motifs

The presence of motifs within the sequences of *fliC* supports the high similarity of its protein sequences. Table 3 below displays the types of motifs found in our sequences.

**Figure 4:**
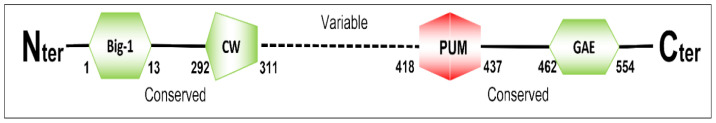
Schematic representation of the distribution of the main motifs found on the *fliC* protein using the PROSITE database

The *fliC* protein has highly conserved N-terminal and C-terminal regions. The N-terminal region contains two motifs called Big-1 and CW, while the C-terminal region contains the motifs PUM and GAE. No motifs are found in the core region (Figure 4).

#### Phylogeny of the fliC Gene

**Figure 5:**
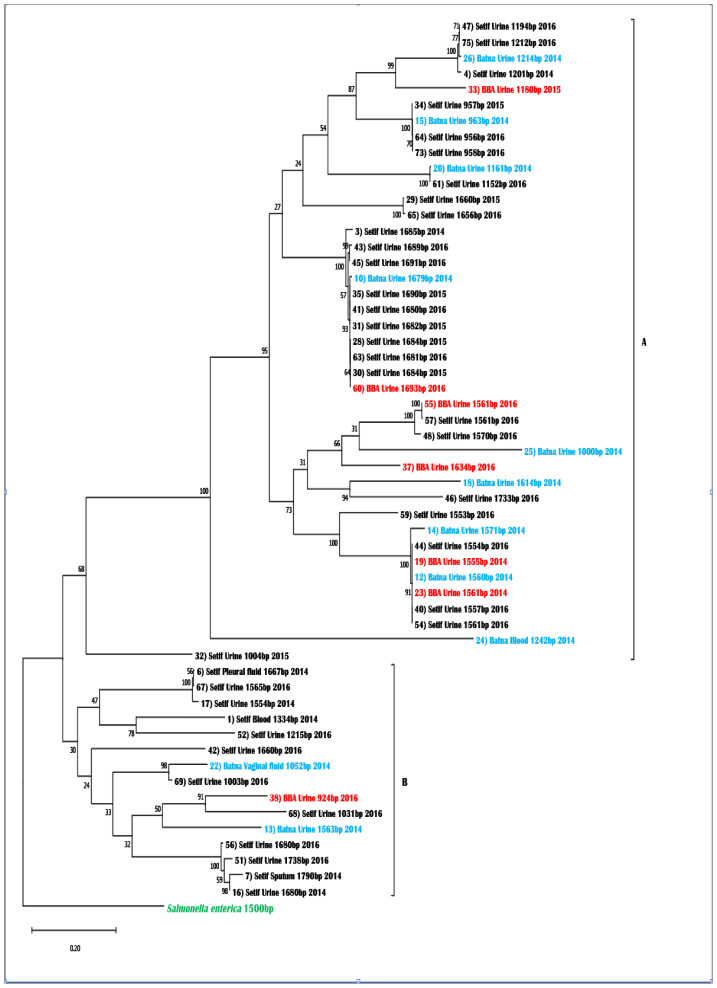
Phylogenetic tree of the *fliC* gene from the pathogen *E. coli* rooted by the *fliC* gene of *S. enterica*

The nucleotide sequence of the phase 1 flagellin of *S. enterica Typhimurium* was used to construct a phylogenetic tree. This tree shows two main groups of *E. coli fliC* alleles: cluster A and cluster B (Figure 5).

The phylogram topology revealed two primary clades originating from the root. Clade A had 41 OTUs (operational taxonomic units), while clade B had 15 OTUs. Clade A was homogeneous, except for one OTU (Setif, N °32) that left the group. The remaining isolates *(n* = 40) were divided into two groups with a bootstrap value of 95%. The OTUs had a wider distribution in the Province of Setif than in the Provinces of Bordj Bou Arreridj and Batna. The Setif Province had a greater number of OTUs, while the Bordj Bou Arreridj and Batna Provinces had a balanced distribution with 6 and 9 OTUs, respectively. The number of OTUs in the 2014 and 2015 collections was relatively balanced, with 13 and 8, respectively. However, the 2016 collection had 20 OTUs. Notably, urinary tract infections were the predominant type in Clade A, accounting for 97.6% of the samples (40 out of 41). There was only one blood sample in Clade A. Based on the nature of the aligned sequences (primary structure), there was a significant similarity among those in Clade A as opposed to sequences from Clade B. The latter showed similarities with other *E. coli* strains from samples other than urine.

Clade B was divided into two subclades with a 30% bootstrap value. It is mainly composed of OTUs from Setif Province, with only one individual from Bordj Bou Arreridj Province and two from Batna Province. Additionally, this clade was distinguished by the complete absence of OTUs collected in 2015, while there was a balanced presence of OTUs from 2014 and 2016. Urine samples clearly represented Clade B compared to other samples, such as blood, sputum and pleural fluid.

The gene phylogeny for *E. coli fliC* is rooted in the *fliC gene* of *S. enterica Typhimurium*. The maximum likelihood method and Kimura 2-parameter model were used to infer the evolutionary history. The tree with the highest log likelihood of −34242.14 was observed. The percentage of trees with clustered associated taxa is indicated next to the branches. The initial tree(s) for the heuristic search were automatically obtained by applying the neighbour-joining and BioNJ algorithms to a matrix of pairwise distances. These distances were estimated using the maximum composite likelihood (MCL) approach. The topology with the highest log likelihood value was selected. The tree is drawn to scale, with branch lengths measured in substitutions per site. The analysis included 57 nucleotide sequences. The final dataset contained 2,824 positions. MEGA11 was used for evolutionary analyses. The analysis of the parameters and *E. coli* distribution in this investigation, using multiple correspondence analysis (Supplementary Figure 2 and Table 5), revealed correlations between certain parameters and no correlations in other cases. Therefore, when examining the correlation between the parameters Year and Season, a strong positive correlation of 0.948 was observed. This suggests a significant relationship between these two parameters and the distribution of *E. coli*. Interestingly, there was a weak correlation between season and sex (*r* = 0.011). Based on these findings, it appears that the distribution of *E. coli* is not influenced by the season or sex parameters.[Table t4-12mjms3105_oa]

## Discussion

In this study, the *fliC* genes of *E. coli* strains collected from various sample types and provinces exhibited a remarkable level of similarity in their N-terminal and C-terminal regions. Undoubtedly, the remarkable structural uniformity observed at both the N-terminal and C-terminal ends provides valuable insights into the wide range of structural variations exhibited by flagellin. Nevertheless, the protein’s central region has revealed significant differences that serve as molecular indicators of the varying structural characteristics found in different *E. coli* strains. However, there is notable diversity observed in the central region of these genes. The presence of diverse flagellin gene variations within the core region has been detected in various bacterial species. The flagellin gene has been found to exhibit diversity in several bacterial species. These include *Campylobacter jejuni* (15–17), *Pseudomonas aeruginosa* (18, 19), *S. enterica* (20), *Vibrio parahaemolyticus* (21), *Helicobacter pylori* (22) and *Burkholderia cepacia* (23, 24). These studies have successfully highlighted the notable genetic diversity of the flagellar gene among various bacterial species. The utilisation of flagellin genes as biomarkers can aid in the recognition and understanding of different strains present in a population. In a study conducted by Tasteyre et al. (25), the DNA sequence of *fliC* was analysed in 47 isolates of *Clostridium difficile* belonging to 12 distinct serogroups (C, D and X). The study revealed a notable range of differences in the central domain of the *fliC* gene, but the N- and C-terminal domains were consistent and unchanged.

Multiple sequence alignment analysis revealed significant similarities in both the N- and C-terminal regions, suggesting strong sequence conservation. The conserved regions are likely functional domains within the gene. Several researchers have previously demonstrated the conservation of the two regions in question. Similar findings have been reported by Winstanley and Morgan, Reid et al., Tasteyre et al. and Fereshteh and Badmasti (25–28). These studies suggest that the two conserved regions are highly conserved, while the central region shows variability. The identification of motifs documented in the PROSITE database supports conservation. In this study, we identified two distinct motifs, Big-1 and CW, located at the beginning of the sequences in the N-terminal region. The Big-1 motif is found from residues 1 to 11, and the CW motif is found from residues 292 to 311. Furthermore, we observed two residues, PUM and GAE, in the C-terminal region. The PUM residues are located from residues 418 to 437 and the GAE residues are found from residues 462 to 554. These findings offer valuable insights into the structural characteristics of proteins. Beutin et al. (29) analysed the *fliC* gene sequence of the P12b strain and compared it to the *fliC* sequences of five other *E. coli* H4 or H17 strains. The analysis showed that the sequences at both the DNA and amino acid levels were significantly similar, indicating strong conservation of this gene. Amhaz et al. (30) conducted a study using RFLP-PCR *fliC* to examine the genetic diversity of major enteroinvasive *E. coli* serotypes. The research findings showed that the serotypes had limited diversity in the *fliC* gene, especially in its central region.

Fereshteh and Badmasti (28) recently conducted a study on the multiple alignment of 392 non-redundant whole *fliC* protein sequences from Enterobacteriaceae. Through analysis, two conserved sections were identified, the D0 and D1 domains, along with one hypervariable region called the *fliC* protein core region. These findings provide insights into the structural characteristics of *fliC* proteins in the Enterobacteriaceae family. This study examined the sequence heterogeneity and diversity of *S. enterica subsp. enterica fliC* serovars. The results revealed significant variation among the serovars, suggesting a diverse genetic landscape. Distance matrix analysis revealed similarities between *E. coli fliC* proteins and *S. enterica fliC* proteins. This study revealed that *fliC* is a reliable marker for distinguishing between different members of the Enterobacteriaceae family. Reid et al. (27) investigated the genetic characteristics of pathogenic *E. coli* strains by directly sequencing the *fliC* gene. The *fliC* gene encodes the H antigen, which is important for the pathogenicity of *E. coli*. Reid et al. (27) reported that the N- and C-terminal regions of *fliC* gene alleles are highly conserved among different pathogenic *E. coli* serotypes. These regions are functionally important and likely contribute to the overall structure and function of the H antigen. The central part of the *fliC* gene varies significantly among different pathogenic *E. coli* serotypes, exhibiting numerous variations. This suggests that the central region experiences selective pressures that lead to genetic variation and adaptation in these strains. The study by Reid et al. (27) offers valuable insights into the genetic diversity of pathogenic *E. coli* strains, specifically regarding the *fliC* gene. Additional research in this field could provide insights into the specific mechanisms that contribute to the polymorphism and diversity observed in the central region of the *fliC* gene. Understanding these mechanisms could help us better understand the pathogenicity of different *E. coli* serotypes.

Polysaccharides O and flagellin are the main antigens of Gram-negative bacteria and are also referred to as the O and H antigens. Both antigens are highly polymorphic (31). There are 53 types of *E. coli* flagellar H-types (H1–H56) composed of polymerised flagellin subunits. However, H13, H22 and H50 are no longer present. Previous research has shown that genes in the *fliC* locus and other loci encode 43 out of the 53 H-antigen types in *E. coli* (31–34). The *fliC* sequences of 43 H-types were compared, revealing distinct sequence features. The core region showed high variability, while the 5′ and 3′ regions were conserved (> 90%). The core sections had less than 90% similarity (31). The diversity and conservation of sequences in the *fliC* and non*fliC* genes enable the molecular identification of *E. coli* H-types (35). The *E. coli* isolates from the Setif Province in 2016 were found in urine samples collected during the spring and summer seasons. They were detected in individuals of all age groups and both sexes. Therefore, we can conclude that certain relationships, such as the correlation between spring and urine samples, impact the dispersion of *E. coli*. Bacteria were more common in the urine of patients during the spring and summer. Pathogens in the Bordj Bou Arreridj Province were linked to the year 2016, urine samples, the spring and summer seasons, the two age groups (1 year old–19 years old and 19 years old–37 years old) and the female sex. Batna Province was associated with the 2014 collection, blood samples, the winter season and the age group (55 years old–73 years old). Female gender was more closely associated with this province than male gender.

The current investigation examined the *fliC* gene to determine the results of phylogram analysis. The analysis aimed to identify patterns or preferences in the distribution of *E. coli* isolates based on their spatiotemporal characteristics. The findings show that the clades in the phylogram are diverse and do not have any significant similarities in terms of time or space. The observed outcome suggested that the *fliC* gene sequences are very similar, indicating that no mutations could affect the gene’s function. The lack of mutations may be due to factors such as environmental conditions, sample nature or the short time between collection and analysis. The high similarity in the *fliC* gene sequences of all the *E. coli* strains in this study indicates significant structural and functional similarities among them. These findings indicate that the *fliC* gene is highly conserved among the isolated strains.

This study revealed a very high positive significant correlation (*r* = 0.948; critical *r* = 0.2830 at *P* = 0.01) between the variables of season and year of sampling. The data analysis showed a strong association between these two factors. This finding suggests a strong correlation between the season of sample collection and the year of sample acquisition. The correlation coefficient of 0.948 suggests a strong linear relationship between the variables, providing additional evidence for the observed connection. These results enhance our understanding of how seasonal variations and the year of sampling interact, providing insight into how these factors may influence the phenomenon being studied. Our research revealed a correlation coefficient of 0.595 between the sampling year and regional factors. This finding suggests a relationship between the two variables, indicating that changes in the sampling year may be associated with variations in the region. Additional investigations are needed to comprehend the underlying factors that contribute to this correlation and its implications for our study.

## Conclusion

We analysed *fliC* protein sequences to study *fliC* mutants in hospitals across three Algerian Provinces. Our findings confirm the complete absence of *fliC* mutants in these healthcare facilities. The use of the *fliC* gene as a marker has great potential for studying the genetic diversity of *E. coli* isolates from clinical samples.

## Limitations of the Study

While our study sheds light on the diversity of the *fliC* gene in *E. coli* and the absence of mutant strains, the relatively small sample size highlights the importance of cautious interpretation. Future studies with larger and more diverse sample sizes are required to confirm and expand our findings. In addition, due to the nature of the study design, we cannot establish an infinite causality between exposures and outcomes, nor can we control confounding variables such as genetic predisposition to *E. coli* infection.

## Figures and Tables

**Table 1:** Primers used for PCR-STD reactions

Target gene	Type of PCR	Primer	5′-3′ sequence	Size (bp)
16S	PCR-STD	16S rDNA-F	AGAGTTTGATCCTGGCTCAG	1,000–1,500
rDNA		16S rDNA-R	GGCTACCTTGTTACGACT	
fliC	PCR-STD	*fliC-F*	ATGGCACAAGTCATTAATACCAACA	1,000–2,000
		*fliC-R*	TTAACCCTGCAGCAGAGACAGA	

**Table 2:** Distribution of bacterial numbers by age group, sex, sample type, year of isolation, season of isolation and geographic regions

Parameters	*n*	%
Age group (years old)		
≤ 14	17	22.67
15–29	16	21.33
30–59	25	33.33
≥ 60	17	22.67
Sex		
Male	23	30.67
Female	52	69.33
Type of samples		
Blood	2	2.67
Urine	69	92.00
Plural fluid	2	2.67
Vaginal fluid	1	1.33
Sputum	1	1.33
Year of isolation		
2014	26	34.67
2015	9	12.00
2016	40	53.33
Geographical region		
Sétif	54	72.00
Batna	13	17.33
Bordj Bouarreridj	8	10.67
Type of seasons		
Winter	23	30.67
Autumn	12	16.00
Spring	19	25.33
Summer	21	28.00

**Table 3:** Types and characteristics of *fliC* gene protein motifs within our sequences from PROSITE database

Motif	Code	Position	Sequence	Score	Sample source	Geographic location
Big-1 (bacterial Ig-like domain 1) domain profile	PS51127	1–13	MAQVINTNSLSLI	5.099	Blood, urine, sputum, pleural fluid, genital	Setif Batna
CW Cell wall-binding repeat profile	PS51170	292–311	ANGKITIGGQEAY LTSDGNL	5.955	Urine, sputum	Setif, Batna, Bordj Bou Arreridj Setif
GAE Gamma-adaptin ear (GAE) domain profile	PS50180	462–554	ATTNPLAALDDAIASID KFRSSlgaiqNRLDSAVT NLNTTTNLSEAQsrIQD ADYATEVSNMSKAQII QQAGNSVLAKANQVPQ QVLSLQG	6.561	Urine, Pleural fluid	Setif
PUM Pumilio RNA-binding repeat profile	PS50302	418–437	EVSNMSKAQIIQQAGN SVLQ	4.744	Urine	Bordj Bou Arreridj

**Table 4 t4-12mjms3105_oa:** Summary table of the spatial and temporal distribution of the *E. coli* pathogen

Province	Year of collection	Season	Type of sample	Gender	Age group (years old)
Setif	2016	Spring + Summer	Urine	M + F	All age groups
Bordj Bou Arreridj	2016	Spring + Summer	Urine	F	All age groups
Batna	2014	Winter	Blood	F	All age groups

Notes: M= male, F = female

**Table 5:** Values of the correlation coefficients

Variables	Age	Gender	Collection	Season	Region	Year
Age	1.000					
Gender	0.066	1.000				
Sample Type	0.191	0.022	1.000			
Season	0.226	0.011	0.344	1.000		
Region	0.045	0.077	0.163	0.461	1.000	
Year	0.289	−0.013	0.380	0.948	0.595	1.000

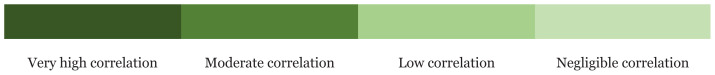

## Appendix

**Supplementary Table 1:** Pairs of *fliC* sequences of very strong identity with its distance score values

Number	Pairs of sequences	Distance score
1	5–6	0.30
2	2–7	2.68
3	8–10	0.24
4	7–11	2.80
5	2–11	0.11
6	20–21	0.69
7	9–22	0.29
8	12–27	0.06
9	19–36	0.00
10	35–39	0.06
11	40–49	0.00
12	41–53	0.77
13	50–55	0.00
14	49–58	0.06
15	26–62	1.24
16	30–66	0.06
17	18–70	6.22
18	39–71	0.06
19	35–71	0.00
20	60–72	0.18
21	45–74	0.71

**Supplementary Table 2:**

Samples	Sex	Age	Sampling type	Collection season	Geographic region	*fliC* (bp)	Year
1	F	65	Blood	Automn	Sétif	1334	2014
2	M	12	Urine	Automn	Sétif	1864	2014
3	F	38	Urine	Automn	Sétif	1685	2014
4	F	66	Urine	Automn	Sétif	1201	2014
5	M	71	Urine	Automn	Sétif	1667	2014
6	M	54	Pleural Fluid	Automn	Sétif	1667	2014
7	F	59	Sputum	Automn	Sétif	1790	2014
8	M	61	Urine	Automn	Sétif	1675	2014
9	M	45	Pleural Fluid	Automn	Sétif	1052	2014
10	F	16	Urine	Automn	Batna	1679	2014
11	F	50	Urine	Automn	Batna	1820	2014
12	M	59	Urine	Winter	Batna	1560	2014
13	M	73	Urine	Automn	Batna	1563	2014
14	F	13	Urine	Winter	Batna	1571	2014
15	F	19	Urine	Winter	Batna	963	2014
16	F	60	Urine	Winter	Sétif	1680	2014
17	F	71	Urine	Winter	Sétif	1554	2014
18	M	46	Urine	Winter	Batna	1614	2014
**19**	**F**	**53**	**Urine**	**Winter**	**Bordj Bouarreridj**	**1555**	**2014**
20	F	47	Urine	Winter	Batna	1161	2014
21	F	11	Urine	Winter	Batna	1154	2014
22	F	24	Vaginal Fluid	Winter	Batna	1052	2014
**23**	**M**	**29**	**Urine**	**Winter**	**Bordj Bouarreridj**	**1561**	**2014**
24	F	44	Blood	Winter	Batna	1242	2014
25	F	55	Urine	Winter	Batna	1000	2014
26	F	67	Urine	Winter	Batna	1214	2014
27	M	17	Urine	Winter	Sétif	1568	2015
28	F	45	Urine	Winter	Sétif	1684	2015
29	F	8	Urine	Winter	Sétif	1660	2015
30	F	26	Urine	Winter	Sétif	1684	2015
31	F	25	Urine	Winter	Sétif	1682	2015
32	F	17	Urine	Winter	Sétif	1004	2015
**33**	**F**	**18**	**Urine**	**Winter**	**Bordj Bouarreridj**	**1180**	**2015**
34	F	63	Urine	Winter	Sétif	957	2015
35	F	8	Urine	Winter	Sétif	1690	2015
36	F	29	Urine	Spring	Bordj Bouarreridj	1557	2016
37	M	5	Urine	Spring	Bordj Bouarreridj	1634	2016
38	M	37	Urine	Spring	Bordj Bouarreridj	924	2016
39	M	1	Urine	Spring	Sétif	1682	2016
40	F	38	Urine	Spring	Sétif	1557	2016
41	F	3	Urine	Spring	Sétif	1680	2016
42	M	75	Urine	Spring	Sétif	1660	2016
43	F	47	Urine	Spring	Sétif	1689	2016
44	F	26	Urine	Spring	Sétif	1554	2016
45	M	8	Urine	Spring	Sétif	1691	2016
46	F	88	Urine	Spring	Sétif	1733	2016
47	F	22	Urine	Spring	Sétif	1194	2016
48	F	44	Urine	Spring	Sétif	1570	2016
49	M	64	Urine	Spring	Sétif	1559	2016
50	M	4	Urine	Spring	Sétif	1561	2016
51	F	60	Urine	Spring	Sétif	1738	2016
52	F	29	Urine	Spring	Sétif	1215	2016
53	F	16	Urine	Spring	Sétif	1682	2016
54	F	4	Urine	Spring	Sétif	1561	2016
**55**	**F**	**62**	**Urine**	**Summer**	**Bordj Bouarreridj**	**1561**	**2016**
56	F	74	Urine	Summer	Sétif	1680	2016
57	F	10	Urine	Summer	Sétif	1561	2016
58	F	42	Urine	Summer	Sétif	1558	2016
59	F	1	Urine	Summer	Sétif	1553	2016
**60**	**F**	**8**	**Urine**	**Summer**	**Bordj Bouarreridj**	**1693**	**2016**
61	F	5	Urine	Summer	Sétif	1152	2016
62	M	42	Urine	Summer	Sétif	1207	2016
63	M	30	Urine	Summer	Sétif	1681	2016
64	F	65	Urine	Summer	Sétif	956	2016
65	F	45	Urine	Summer	Sétif	1656	2016
66	M	60	Urine	Summer	Sétif	1675	2016
67	F	56	Urine	Summer	Sétif	1565	2016
68	F	33	Urine	Summer	Sétif	1031	2016
69	M	21	Urine	Summer	Sétif	1003	2016
70	F	45	Urine	Summer	Sétif	1609	2016
71	M	9	Urine	Summer	Sétif	1685	2016
72	F	43	Urine	Summer	Sétif	1685	2016
73	M	1	Urine	Summer	Sétif	958	2016
74	F	51	Urine	Summer	Sétif	1685	2016
75	F	18	Urine	Summer	Sétif	1212	2016

**Supplementary Table 3:** List of accession numbers of the DNA sequences of our *fliC* gene of interest, plus the results of its analysis using the BLAST programme against the GenBank sequences

Samples	Accession numbers	% Cover	e-Value	% Id	Blast results
1	OP600171	99	0.0	100	WP_000079774.1
2	OP600172	99	0.0	100	WP_000079699.1
3	OP600173	99	0.0	100	EFC4190305.1
4	OP600174	99	0.0	100	MBS8541439.1
5	OP600175	99	0.0	100	WP_000079726.1
6	OP600176	81	0.0	100	WP_235639195.1
7	OP600177	99	0.0	100	WP_001696043.1
8	OP600178	97	0.0	99.63	WP_044074870.1
9	OP600179	100	0.0	99.71	HAM5355263.1
10	OP600180	99	0.0	99.28	EHS6069984.1
11	OP600181	99	0.0	99.5	EGK3893988.1
12	OP600182	100	0.0	100	AAF85764.1
13	OP600183	96	0.0	100	WP_218792500.1
14	OP600184	29	3e–93	96.18	UKM08107.1
15	OP600185	99	1e–176	100	HAM5355263.1
16	OP600186	97	0.0	100	HBA1462600.1
17	OP600187	97	0.0	100	HAI5444722.1
18	OP600188	99	0.0	100	WP_032083558.1
19	OP600189	96	0.0	100	WP_149857796.1
20	OP600190	98	0.0	100	EFA5241419.1
21	OP600191	94	0.0	100	EFD7673086.1
22	OP600192	99	0.0	99.71	WP_113248328.1
23	OP600193	96	0.0	100	HAJ6334821.1
24	OP600194	85	0.0	100	EFE8354926.1
25	OP600195	98	1e–151	96.84	EHS6082838.1
26	OP600196	99%	0.0	100.00%	WP_113495836.1
27	OP600197	99%	0.0	99.81%	AAF85764.1
28	OP600198	99%	0.0	100.00%	WP_044074870.1
29	OP600199	99%	0.0	100.00%	WP_000079688.1
30	OP600200	99%	0.0	100.00%	OWF26411.1
31	OP600201	99%	0.0	100.00%	HBA1462600.1
32	OP600202	92%	1e–171	99.35%	MBE0881690.1
33	OP600203	99%	0.0	100.00%	WP_032342875.1
34	OP600204	98%	3e–174	99.68%	EFC9733409.1
35	OP600205	99%	0.0	100.00%	EHS6069984.1
36	OP600206	96%	0.0	100.00%	MBK1749717.1
37	OP600207	99%	0.0	100.00%	WP_000079707.1
38	OP600208	66%	1e–113	98.54%	MBW2857713.1
39	OP600209	99%	0.0	100.00%	EHS6069984.1
40	OP600210	96%	0.0	100.00%	WP_149857796.1
41	OP600211	96%	0.0	100.00%	WP_044074870.1
42	OP600212	99%	0.0	100.00%	WP_000079688.1
43	OP600213	92%	2e–147	99.64%	WP_044074870.1
44	OP600214	96%	0.0	100.00%	WP_149857796.1
45	OP600215	99%	0.0	100.00%	WP_021580819.1
46	OP600216	99%	0.0	100.00%	MBS9183088.1
47	OP600217	99%	0.0	100.00%	WP_113495836.1
48	OP600218	99%	0.0	100.00%	WP_000079703.1
49	OP600219	99%	0.0	100.00%	HAJ6334821.1
50	OP600220	96%	0.0	100.00%	WP_218792500.1
51	OP600221	92%	0.0	100.00%	OWF26411.1
52	OP600222	100%	0.0	99.75%	MBS8541439.1
53	OP600223	99%	0.0	99.82%	WP_021580819.1
54	OP600224	99%	0.0	100.00%	WP_149857796.1
55	OP600225	96%	0.0	100.00%	WP_218792500.1
56	OP600226	97%	0.0	97.13%	WP_044074870.1
57	OP600227	99%	0.0	100.00%	WP_218792500.1
58	OP600228	96%	0.0	100.00%	HAI5444722.1
59	OP600229	99%	0.0	100.00%	WP_000079693.1
60	OP600230	99%	0.0	100.00%	WP_249577752.1
61	OP600231	100%	0.0	100.00%	EFA5241419.1
62	OP600232	99%	0.0	100.00%	MBS8541439.1
63	OP600233	99%	0.0	100.00%	WP_044074870.1
64	OP600234	99%	3e–176	100.00%	WP_124809510.1
65	OP600235	99%	0.0	100.00%	WP_113241545.1
66	OP600236	96%	0.0	100.00%	WP_044074870.1
67	OP600237	99%	0.0	100.00%	AAF85764.1
68	OP600238	84%	5e–108	92.09%	MCK3588766.1
69	OP600239	98%	9e–122	92.12%	HAJ2083076.1
70	OP600240	99%	0.0	100.00%	WP_001759232.1
71	OP600241	99%	0.0	100.00%	WP_249577752.1
72	OP600242	99%	0.0	100.00%	WP_044074870.1
73	OP600243	99%	6e–177	100.00%	HAJ8822207.1
74	OP600244	99%	0.0	100.00%	WP_044074870.1
75	OP600245	99%	0.0	100.00%	HAN7351110.1

**Supplementary Table 4:** Distance Matrix *fliC* 75

Distance Matrix	Column																
Uncorrected for Multiple Substitutions																	
Using base positions 123 in the codon																	
Gap weighting is 0.000000																	
	1	2	3	4	5	6	7	8	9	10	11	12	13	14	15	16	17
	0	46.92	75.9	74.94	48.2	48.27	47.07	75.53	41.81	75.53	47	74.02	76.05	72.15	75.39	70.05	74.25
		0	76.26	75.77	49.67	49.55	2.68	76	42.57	76.12	0.11	76.35	77.61	73.46	74.25	69.46	76.25
			0	74.85	74.77	74.65	76.5	76.12	76.67	76.24	76.32	69.87	75.62	74.86	70.09	74.64	76.64
				0	76.35	76.44	75.52	77.1	76.29	77.02	75.6	78.02	74.6	62.53	78.4	73.77	74.77
					0	0.3	50.03	77.42	44.38	77.42	49.79	73.4	77.22	73.27	75.08	69.91	77.8
						0	49.91	77.42	44.29	77.42	49.67	73.4	77.22	73.33	74.97	70.03	77.8
							0	75.94	43.33	75.94	2.8	76.15	77.35	73.14	74.56	69.52	75.74
								0	77.71	0.24	76.06	77.56	73.51	76.13	75.08	78.09	72.84
									0	77.9	42.67	74.1	76.1	73.05	75.18	70.1	73.24
										0	76.18	77.69	73.58	76.19	75.08	78.08	72.91
											0	76.35	77.54	73.39	74.35	69.4	76.25
												0	76.28	75.19	71.03	74.17	74.58
													0	75.18	75.49	77.8	69.5
														0	79.54	73.65	75.1
															0	75.18	76.53
																0	76.83
																	0

Distance Matrix	Column															
Uncorrected for Multiple Substitutions																
Using base positions 123 in the codon																
Gap weighting is 0.000000																
	18	19	20	21	22	23	24	25	26	27	28	29	30	31	32	33
	72.75	77.55	74.33	74.52	41.9	72.15	73.16	74.6	75.12	73.95	73.65	75.3	74.1	74.02	76.69	73.47
	72.8	78.26	74.68	74.52	42.48	74.18	72.13	74.7	74.55	76.28	74.94	77.35	74.52	75.21	75.3	72.88
	71.07	75.56	75.88	75.82	76.67	68.35	72.98	76.7	73.23	69.71	80.17	76.93	76.37	77.65	77.29	71.19
	76.44	76.27	71.49	71.14	76.19	76.19	77.79	67.9	77.52	78.02	73.19	77.19	72.19	72.86	73.51	75.59
	74.23	77.17	76.06	75.82	44.48	73.54	73.67	74.2	74.05	73.28	73.87	75.66	72.85	73.57	77.09	74.15
	74.16	77.17	75.88	75.65	44.38	73.61	73.58	74.2	74.05	73.28	73.87	75.72	72.73	73.63	77.19	74.07
	73.05	77.62	75.02	74.87	43.24	73.93	71.7	73.9	74.22	76.08	75.06	77.23	74.7	74.97	74.8	73.22
	77.14	69.9	77.35	77.64	77.81	76.43	75.64	77.3	78.75	77.61	76.3	70.48	79.94	77.97	74.9	75.51
	74.38	76.38	76.67	76.67	0.29	73.52	70.95	73.6	72.86	74.1	73.43	76.57	75.81	75.24	75.8	74.95
	77.2	69.97	77.35	77.64	78	76.43	75.73	76.9	78.91	77.74	76.24	70.3	79.81	77.72	74.9	75.51
	72.68	78.33	74.68	74.52	42.57	74.25	72.3	74.6	74.38	76.28	75.06	77.29	74.41	75.15	75.3	73.05
	60.58	79.36	75.8	76	74	72.12	68.95	77.5	61.04	0.06	75.45	76.73	78.08	78.14	78.19	70
	78.5	73.44	70.2	70.36	76.1	76.68	74.96	75.8	76.94	76.39	74.79	73.77	73.45	73.51	72.41	76.86
	77.78	75.88	72.27	72.18	72.95	53.43	74.44	70.5	77.27	75.26	72.95	77.78	71.42	72.06	72.61	74.83
	70.2	76.43	76.22	76.12	75.08	69.57	69.89	77.99	70.61	70.92	76.74	79.02	78.61	78.5	77.26	69.57
	72.43	76.85	75.88	75.65	70.38	73.03	73.58	74.4	74.22	74.23	71.85	76.99	74.11	75.06	75.7	74.49
	78.31	72.97	73.9	73.66	73.24	78.31	75.3	77.2	74.71	74.52	74.26	73.75	73.94	73.49	72.21	75.51
	0	78.2	75.8	75.82	74.19	69.51	69.13	79.2	60.38	60.65	77.2	78.19	75.84	77.57	78.69	70.08
		0	75.54	75.74	76.19	78.52	78.56	75.6	78.34	79.42	78.07	70.42	77.81	76.59	73.21	76.02
			0	0.69	76.57	74.59	75.45	73.2	77.17	75.88	70.03	79.76	74.25	74.42	72.61	75.8
				0	76.57	74.44	75.48	73.2	77.04	76.08	69.76	79.81	74.26	74.61	72.81	75.56
					0	73.43	71.05	73.5	72.95	74	73.52	76.57	75.9	75.33	75.6	74.67
						0	69.9	77.1	67.87	72.2	78.16	76.17	76.55	77.39	75.4	70.17
							0	73.7	73.67	68.95	76.76	76.5	76.59	76.93	76.89	72.9
								0	77	77.6	71.4	75.1	71.8	71.7	72.7	76.8
									0	61.04	76.77	75.95	77.18	77.51	76.69	71.86
										0	75.45	76.85	78.12	78.06	78.19	69.92
											0	77.47	71.56	71.52	74.4	76.69
												0	77.83	75.36	75.4	78.47
													0	29.73	73.01	78.05
														0	73.01	79.24
															0	77.69
																0

Distance Matrix	Column															
Uncorrected for Multiple Substitutions																
Using base positions 123 in the codon																
Gap weighting is 0.000000																
	34	35	36	37	38	39	40	41	42	43	44	45	46	47	48	49
	76.49	77.25	77.55	74.47	75.54	77.25	72.82	73.12	77.33	74.47	75.6	75	74.1	76.8	75	72.82
	73.98	79.23	78.29	72.95	76.52	79.19	74.44	74.94	76.69	74.19	75.93	74.63	75.3	76.3	76.88	74.47
	75.97	74.18	75.53	75.95	75.87	74.14	77.65	77.14	73.67	72.17	76.38	70.15	76.56	80.23	75.54	77.55
	57.99	76.44	76.27	71.19	74.57	76.44	71.69	71.19	76.35	76.94	77.6	76.44	71.86	78.06	77.6	71.69
	77.22	77.48	77.14	74.6	77.71	77.48	75.21	75.2	76.63	74.47	75.48	74.71	72.37	76.8	77.07	75.24
	77.32	77.3	77.14	74.6	77.71	77.3	75.4	75.32	76.57	74.53	75.55	74.65	72.61	76.88	77.07	75.43
	74.82	78.46	77.65	72.89	76.3	78.42	74.44	74.7	76.75	74.07	76.19	74.33	75.13	75.96	77.07	74.47
	78.06	71.52	69.94	76.74	74.03	71.58	75.53	74.63	75.48	79.4	59.33	77.43	77.25	71.36	69.3	75.5
	77.53	77.52	76.38	74.86	78.35	77.52	73.05	75.52	76.86	76.67	74.95	76.19	74.19	76.95	77.62	73.05
	77.95	71.53	70.01	76.62	73.92	71.59	75.47	74.75	75.42	79.45	59.27	77.55	77.25	71.52	69.43	75.43
	73.88	79.23	78.36	73.01	76.52	79.19	74.44	74.94	76.57	74.19	76	74.63	75.3	76.21	76.82	74.47
	76.8	78.14	79.32	77.24	75.54	78.14	76.62	76.67	72.69	57.88	78.51	59.36	78.4	75.88	74.42	76.59
	75.97	71.79	73.47	75.05	76.08	71.79	75.34	73.77	78.37	77.42	76.06	76.33	72.74	76.3	73.83	75.3
	55.8	76.07	75.92	74.03	77.71	76.07	63.58	71.61	73.14	75.68	77.86	74.54	72.82	77.39	74.39	63.57
	76.38	73.94	76.43	77.36	75.32	73.94	77.88	75.29	74.66	72.38	76.12	72.07	78.09	79.02	73.94	77.88
	75.97	77.08	76.88	72.83	78.57	77.08	72.58	73.1	76.2	73.39	76.83	72.26	74.46	73.79	75.99	72.61
	74.29	73.62	72.97	74.45	79.44	73.62	73.42	72.84	76.32	75.74	72.59	75.93	72.91	75.21	73.42	73.42
	75.76	79.06	78.16	76.27	75.65	79.06	77.01	77.32	72.74	61.65	76.77	60.16	76.52	75.88	76.43	77.04
	76.59	59.23	0	76.98	73.27	59.16	76.59	75.95	73.5	79.23	72.14	77.88	74.86	71.11	71.32	76.59
	70.64	75.71	75.54	60.55	76.41	75.62	60.64	60.64	75.28	75.97	77.52	76.66	62.27	76.57	77.86	60.64
	70.53	76.08	75.74	60.4	76.52	76.17	60.14	60.4	75.22	75.82	77.47	76.6	61.79	76.6	78.08	60.14
	77.53	77.52	76.19	74.95	78.57	77.52	73.14	75.43	76.67	76.57	74.95	76.1	74.29	76.76	77.52	73.14
	76.8	77.83	78.55	77.83	72.29	77.83	75.4	75.59	73.22	71.17	77.16	70.08	78.28	77.97	77.32	75.37
	74.82	74.96	78.56	75.13	75.97	74.96	74.87	75.47	73.41	72.56	77.1	71.18	76.07	77.44	77.44	74.87
	72.73	74	75.6	73.1	75.43	73.9	70.3	71.8	77.2	78.5	77.2	80.1	72	75.9	75.4	70.3
	78.27	76.77	78.34	77.1	73.05	76.77	77.68	76.85	74.79	59.39	78.58	60.13	77.27	76.8	74.63	77.68
	76.91	78	79.38	77.36	75.65	78	76.69	76.66	72.77	57.78	78.51	59.38	78.44	75.96	74.36	76.65
	73.15	79.99	78.1	70.26	77.16	79.96	70.01	71.61	75.9	75.3	76.06	74.35	69.89	76.72	76.56	70.04
	77.85	71.02	70.39	79.44	74.35	71.02	80.6	78.19	72.65	76.14	70.53	76.93	77.95	69.85	61.85	80.56
	67.92	77.26	77.71	72.52	78.25	77.29	69.88	70.24	75	75.53	78.06	76.66	71.26	75.63	77.01	69.79
	68.03	75.09	76.56	74.05	78.35	75.09	70.65	72.14	74.58	76.28	74.9	77.82	72.89	74.79	75.16	70.62
	73.77	75.7	73.21	71.12	79.44	75.8	73.71	70.82	77.09	76.89	74	78.39	74.8	73.21	72.81	73.71
	75.03	76.86	76.02	75.25	75.32	76.95	74.66	76.95	75.08	70.59	77.88	69.41	76.44	77.37	76.86	74.66
	0	77.01	76.59	71.37	75.97	77.01	72	70.64	76.07	75.86	76.38	76.28	71.37	78.16	78.27	72
		0	59.22	76.25	76.41	0.06	76.56	76.13	75	77.68	71.81	79.94	74.56	71.44	71.72	76.59
			0	76.94	73.27	59.15	76.62	75.98	73.41	79.25	72.14	77.84	74.89	71.11	71.23	76.62
				0	77.92	76.32	59.99	60.04	76.44	77.48	77.35	76.87	57.65	76.38	78.34	60.04
					0	76.52	77.27	78.9	73.38	76.41	74.89	74.89	80.3	70.56	71.65	77.27
						0	76.62	76.19	75	77.71	71.88	80.02	74.61	71.36	71.72	76.65
							0	59.09	75.47	77.26	75.35	75.92	59.99	77.3	77.39	0
								0	77.23	73.15	76.71	76.19	60.3	76.13	77.58	59.14
									0	75.18	75.68	76.27	76.27	74.96	72.17	75.43
										0	76.71	37	75.61	77.47	76.82	77.29
											0	77.99	76.45	67.84	72.07	75.35
												0	75.75	76.21	76.5	75.95
													0	75.54	78.85	59.97
														0	69.93	77.3
															0	77.42
																0

Distance Matrix	Column															
Uncorrected for Multiple Substitutions																
Using base positions 123 in the codon																
Gap weighting is 0.000000																
	50	51	52	53	54	55	56	57	58	59	60	61	62	63	64	65
	74.92	76.95	72.67	72.97	76.95	74.92	76.43	74.25	72.82	73.95	74.25	73.61	74.71	76.2	77.62	74.85
	74.76	75.6	73.91	75.15	77.39	74.76	73.45	74.38	74.45	75.72	74.6	75.52	74.63	77.87	76.26	74.46
	76.36	75.85	77.53	77.35	78.09	76.36	74.29	77.07	77.54	71.09	76.2	59.55	73.55	80.19	77.3	77.36
	69.53	73.11	73.77	71.11	77.6	69.53	72.94	72.27	71.61	77.44	77.02	76.3	77.6	77.02	75.94	59.53
	73.8	76.34	73.33	75.32	77.9	73.8	72.85	73.73	75.16	73.92	77	74.91	74.38	77.48	78.03	71.56
	73.8	76.28	73.5	75.38	77.71	73.8	72.91	73.73	75.35	73.92	77	74.91	74.38	77.6	78.14	71.56
	74.63	75.72	73.74	74.91	77	74.63	73.27	74.5	74.45	75.72	74.78	75.52	74.3	77.51	76.99	74.34
	75.66	75.64	75.06	74.51	69.7	75.66	74.27	76.55	75.48	76.82	70.57	76.65	78.77	71.52	69.67	77.48
	73.52	74.38	72.1	75.43	76.95	73.52	76.48	74.76	73.05	73.81	74.57	74.76	72.76	75.52	78.03	72.1
	75.46	75.64	75.23	74.63	69.7	75.46	74.39	76.43	75.42	76.88	70.52	76.74	78.94	71.35	69.46	77.29
	74.76	75.55	73.91	75.15	77.32	74.76	73.45	74.38	74.45	75.66	74.54	75.52	74.46	77.81	76.15	74.4
	77.05	74.49	73.74	76.47	76.54	77.05	76.35	77.88	76.64	56.54	76.09	68.66	60.95	76.28	78.24	80.51
	75.4	73.51	75.31	73.7	73.29	75.4	51.31	75.66	75.35	76.69	73	77.26	76.95	73.38	74.58	72.23
	72.97	73.97	75.39	71.74	73.35	72.97	74.6	73.16	63.48	74.95	75.43	74.65	76.78	76.7	74.58	61.62
	79.23	73	73.1	75.18	76.53	79.23	76.01	77.57	77.99	72.48	76.95	72.17	70.72	76.22	78.56	77.99
	73.09	74.05	72.76	73.15	78.22	73.09	74.7	72.84	72.59	72.38	78.93	74.91	74.3	74.88	77.51	74.03
	73.68	72.2	76.95	72.84	71.36	73.68	72.59	73.42	73.36	76.56	73.23	74.48	74.71	72.97	76.46	74.52
	77.07	77.57	73.09	77.2	78.54	77.07	76.52	76.36	76.96	59.18	77.51	72.31	60.2	77.63	77.09	78
	75.05	74.98	76.54	75.82	72.22	75.05	75.63	77.3	76.59	77.46	71.9	74.57	78.28	69.84	71.13	75.5
	61.58	74.16	76.4	60.64	78.21	61.58	73.99	70.11	60.55	76.83	78.29	76.04	77.09	77.17	76.26	72.27
	61.27	73.92	76.52	60.4	78.34	61.27	74.09	70.1	60.05	77.04	78.6	75.95	77.04	77.12	76.26	71.92
	73.52	74.38	72.1	75.33	76.86	73.52	76.48	74.95	73.14	73.52	74.57	74.67	72.86	75.33	78.03	72.29
	77.96	73.99	73.25	75.85	79.24	77.96	76.23	76.62	75.48	68.64	77.96	69.62	67.33	77.51	76.26	77.51
	75.56	75.21	73.67	75.47	75.99	75.56	74.7	76.93	74.79	73.16	76.07	73.87	73.67	76.59	78.35	76.67
	71.2	76.8	74.1	71.6	75	71.2	76.8	70.3	70.4	78	75.4	75.3	77	73.3	75.42	69.4
	77.43	75.29	74.96	76.61	76.36	77.43	76.03	79.32	77.68	58.65	75.78	70.92	1.24	75.21	78.14	78.34
	77.13	74.55	73.74	76.47	76.55	77.13	76.34	77.96	76.7	56.54	76.15	68.66	60.95	76.28	78.24	80.61
	71.94	73.93	73.74	71.4	77.71	71.94	74.17	59.39	69.96	76.43	77.32	78.47	76.53	74.54	74.16	70.47
	78.16	74.52	76.87	78.07	57.08	78.16	75.66	78.16	80.62	77.21	60.54	76.22	75.79	69.52	69.98	75.97
	69.83	75.12	73.5	70.15	76.3	69.83	72.8	71.94	69.77	75.66	78.03	76.65	76.95	76.26	75.42	69.5
	71.36	75.33	72.67	72.29	74.7	71.36	73.63	71.62	70.54	76.37	76.22	76.91	77.2	46.58	75.42	71.92
	73.51	77.09	75.9	71.12	73.41	73.51	74	75.8	73.71	76.59	74.2	75.2	77.09	74.2	74.16	75.1
	76.78	74.66	72.37	76.86	77.37	76.78	75.59	75.93	74.66	71.44	76.78	69.79	71.53	77.8	78.45	77.29
	72.2	76.07	75.55	70.85	77.01	72.2	71.79	72.31	71.89	74.92	78.06	76.38	78.27	76.91	77.51	58.93
	76.49	74.26	75.72	76.04	70.08	76.49	75.65	78.8	76.64	77.98	71.48	75.26	76.7	70.26	72.07	76.15
	75.08	75.02	76.54	75.85	72.19	75.08	75.66	77.33	76.62	77.46	71.93	74.57	78.28	69.81	71.13	75.53
	57.98	76.01	72.92	60.4	78.28	57.98	74.3	68.55	60.08	77.01	76.68	74.22	77.2	76.32	75.84	70.56
	78.68	75.76	76.41	78.79	73.81	78.68	75	76.84	77.38	73.05	73.7	77.71	73.16	73.81	75.11	76.19
	76.55	74.38	75.72	76.1	70.08	76.55	75.65	78.8	76.7	77.98	71.64	75.17	76.7	70.2	72.07	76.15
	61.27	73.22	73.5	59.28	79.38	61.27	73.47	50.48	0.06	77.66	78.16	76.39	77.61	78.1	76.36	70.46
	59.19	76.55	73.99	0.77	78.16	59.19	72.98	71.88	59.18	75.08	80.12	78.21	76.95	76.61	76.88	71.01
	78.28	75.48	76.3	77.41	72.84	78.28	78.07	76.87	75.55	75.6	74.52	77.26	74.63	73.37	75.21	76.87
	76.68	74.96	75.31	73.07	75.78	76.68	74.94	76.68	77.21	58.73	74.72	72.4	59.7	76.5	77.09	77.36
	76.64	71.56	76.21	77.09	69.63	76.64	76.9	75.16	75.29	79.2	71.94	75.78	78.69	70.08	71.23	77.93
	78.03	74.69	76.05	76.04	76.43	78.03	75.06	76.17	75.87	57.12	76.11	71.18	60.53	77.87	76.99	78.02
	58.74	75.36	72.02	60.29	77	58.74	74.7	71.04	60.01	77.78	78.5	75.09	77.45	76.74	75.63	72.22
	75.54	77.55	71.61	76.21	69.77	75.54	74.62	76.97	77.39	73.95	71.61	77.78	76.55	60.39	73.54	76.63
	78.6	76.88	76.71	77.52	60.99	78.6	75.35	75.21	77.41	75.34	58.79	75.61	74.79	68.73	70.19	79.17
	61.19	73.19	73.5	59.33	79.35	61.19	73.44	50.42	0.06	77.66	78.13	76.39	77.61	78.06	76.36	70.37
	0	75.66	72.26	59.32	77.32	0	73.61	70.72	61.17	77.66	78.92	76.04	77.28	76.04	74.06	69.63
		0	74.98	76.81	75.59	75.66	75.3	74.18	73.11	74.24	74.9	75.26	75.12	74.96	74.9	73.43
			0	74.07	76.38	72.26	74.16	73.66	73.42	73.17	76.95	75.61	74.79	74.81	76.67	73.09
				0	78.22	59.32	73.1	72.07	59.37	74.82	79.96	78.04	76.7	76.68	77.09	70.83
					0	77.32	76.3	78.09	79.27	77.21	59.19	76.82	76.29	68.74	69.67	76.68
						0	73.61	70.72	61.17	77.66	78.92	76.04	77.28	76.04	74.06	69.63
							0	73.8	73.43	75.72	74.52	76.39	76.45	75.54	76.05	73.19
								0	50.45	78.17	77.9	77.43	78.94	75.72	75.63	69.44
									0	77.59	78.18	76.48	77.61	78.05	76.46	70.35
										0	77.66	71.27	59.12	77.08	78.45	77.21
											0	79.25	75.79	71.56	68.1	77.11
												0	71.27	77.34	75.21	77.34
													0	74.88	78.03	78.11
														0	69.67	76.93
															0	76.15
																0

Distance Matrix	Column										
Uncorrected for Multiple Substitutions											
Using base positions 123 in the codon											
Gap weighting is 0.000000											
	66	67	68	69	70	71	72	73	74	75	
	74.02	75.3	78.08	74.48	72.37	77.25	74.17	74.11	74.85	75.5	(1)_Setif_Blood_1334bp_2014 1
	74.45	77.32	76.72	75.67	72.16	79.23	74.54	73.8	74.54	75.91	(2)_Setif_Urine_1864bp_2014 2
	76.3	73.04	76.43	76.77	70.79	74.18	76.26	73.8	70.21	76.82	(3)_Setif_Urine_1685bp_2014 3
	72.27	75.02	75.17	72.98	76.77	76.44	76.94	71.19	76.44	76.52	(4)_Setif_Urine_1201bp_2014 4
	72.79	77.76	78.37	75.27	73.77	77.48	76.94	72.55	74.53	76.07	(5)_Setif_Urine_1667bp_2014 5
	72.67	77.64	78.37	75.17	73.71	77.3	76.94	72.55	74.47	76.16	(6)_Setif_Pleural_fluid_1667bp_2014 6
	74.63	77.44	76.82	75.47	72.53	78.46	74.72	74.32	74.36	75.91	(7)_Setif_Sputum_1790bp_2014 7
	79.94	75.14	75.36	76.07	77	71.52	70.51	75.68	77.25	73.02	(8)_Setif_Urine_1675bp_2014 8
	75.71	76.57	77.4	73.08	74.86	77.52	74.48	73.8	76.1	75.9	(9)_Setif_Pleural_fluid_1052bp_2014 9
	79.82	75.02	75.46	76.17	77.13	71.53	70.46	75.68	77.37	73.02	(10)_Batna_Urine_1679bp_2014 10
	74.33	77.25	76.82	75.77	72.16	79.23	74.48	73.8	74.54	75.83	(11)_Batna_Urine_1820bp_2014 11
	78.08	73.72	77.01	76.17	60.13	78.14	75.96	74.43	59.1	77.64	(12)_Batna_Urine_1560bp_2014 12
	73.45	77.35	72.36	74.08	77.16	71.79	73.06	74.43	76.52	73.93	(13)_Batna_Urine_1563bp_2014 13
	71.36	76.17	76.33	73.68	77.98	76.07	75.3	71.61	74.54	73.84	(14)_Batna_Urine_1571bp_2014 14
	78.5	74.14	76.84	76.32	70.4	73.94	77.05	76.3	72.17	77.78	(15)_Batna_Urine_963bp_2014 15
	74.21	75.78	78.56	74.88	72.03	77.08	78.81	75.47	72.5	77.06	(16)_Setif_Urine_1680bp_2014 16
	73.94	76.32	73.52	74.68	77.61	73.62	73.23	75.47	75.8	75.66	(17)_Setif_Urine_1554bp_2014 17
	75.84	75.46	78.86	76.87	6.22	79.06	77.32	77.35	60.04	76.9	(18)_Batna_Urine_1614bp_2014 18
	77.81	73.38	74.78	74.78	78.39	59.23	71.9	78.39	78.07	71.37	(19)_BBA_Urine_1555bp_2014 19
	74.25	76.66	76.43	74.78	77.09	75.71	78.29	69.21	76.66	77.26	(20)_Batna_Urine_1161bp_2014 20
	74.26	76.6	76.24	74.88	76.95	76.08	78.6	69.21	76.6	77.56	(21)_Batna_Urine_1154bp_2014 21
	75.81	76.48	77.4	72.98	74.67	77.52	74.48	74.01	76	75.81	(22)_Batna_Vaginal_fluid_1052bp_2014 22
	76.62	74.5	78.76	76.27	69.96	77.83	78.03	76.51	70.02	77.97	(23)_BBA_Urine_1561bp_2014 23
	76.5	74.44	74.1	74.28	69.38	74.96	76.07	75.05	71.1	77.1	(24)_Batna_Blood_1242bp_2014 24
	71.7	76.2	76.9	75.7	78.4	74	75.3	73.38	80.1	74.8	(25)_Batna_Urine_1000bp_2014 25
	77.18	75.7	76.43	75.57	60.54	76.77	75.62	76.1	60.21	76.4	(26)_Batna_Urine_1214bp_2014 26
	78.12	73.61	77.01	76.17	60.2	78	76.02	74.32	59.12	77.56	(27)_Setif_Urine_1568bp_2015 27
	71.7	75.65	76.92	72.68	77	79.99	77.32	72.13	74.52	78.88	(28)_Setif_Urine_1684bp_2015 28
	77.89	72.14	74.39	74.18	77.81	71.02	60.42	77.24	77.11	59.82	(29)_Setif_Urine_1660bp_2015 29
	0.06	75.78	74.2	72.88	75.33	77.26	78.03	69.52	76.6	75.25	(30)_Setif_Urine_1684bp_2015 30
	29.43	74.76	74.1	72.78	76.94	75.09	76.22	69.52	78	74.5	(31)_Setif_Urine_1682bp_2015 31
	73.01	76.69	75.7	74.38	77.89	75.7	74.2	74.63	78.59	74.3	(32)_Setif_Urine_1004bp_2015 32
	77.97	76.19	75.36	75.27	69.75	76.86	76.86	75.99	69.24	77.29	(33)_BBA_Urine_1180bp_2015 33
	68.03	76.49	75.13	74.61	75.65	77.01	78.06	71.79	76.28	76.59	(34)_Setif_Urine_957bp_2015 34
	77.25	74.06	74.59	75.87	79.55	0	71.51	77.04	80	71.12	(35)_Setif_Urine_1690bp_2015 35
	77.71	73.41	74.78	74.78	78.36	59.22	71.93	78.39	78.03	71.37	(36)_BBA_Urine_1557bp_2016 36
	72.46	78.4	77.21	77.17	76.01	76.25	76.62	69.83	76.87	77.48	(37)_BBA_Urine_1634bp_2016 37
	78.35	69.91	75.76	76.95	75.87	76.41	73.7	71.32	74.78	74.68	(38)_BBA_Urine_924bp_2016 38
	77.25	74.06	74.68	75.77	79.55	0.06	71.64	77.04	80.02	71.12	(39)_Setif_Urine_1682bp_2016 39
	69.88	75.98	76.33	71.98	77.33	76.56	78.1	69.83	75.98	78.3	(40)_Setif_Urine_1557bp_2016 40
	70.33	76.55	76.72	73.38	77.07	76.13	80.06	68.27	76.19	76.82	(41)_Setif_Urine_1680bp_2016 41
	75	50.86	77.59	75.27	72.34	75	74.52	72.34	75.96	74.09	(42)_Setif_Urine_1660bp_2016 42
	75.58	73.93	77.4	76.97	61.65	77.69	74.6	74.53	37.21	76.16	(43)_Setif_Urine_1689bp_2016 43
	78.06	74.84	75.07	76.07	76.38	71.81	71.88	78.18	77.73	71.04	(44)_Setif_Urine_1554bp_2016 44
	76.78	75.02	78.18	77.37	59.54	80	75.96	75.68	0.71	76.65	(45)_Setif_Urine_1691bp_2016 45
	71.16	77.83	76.43	73.98	76.76	74.6	78.34	70.88	76.14	77.81	(46)_Setif_Urine_1733bp_2016 46
	75.54	73.79	73.91	75.67	75.88	71.44	71.52	75.16	76.38	73.53	(47)_Setif_Urine_1194bp_2016 47
	77.07	72.2	76.62	77.77	76.31	71.72	58.66	75.05	76.37	60.73	(48)_Setif_Urine_1570bp_2016 48
	69.79	76.01	76.33	71.98	77.36	76.59	78.06	69.83	76.01	78.3	(49)_Setif_Urine_1559bp_2016 49
	69.76	75.14	77.69	75.37	77.83	76.49	78.86	70.56	77.83	78.96	(50)_Setif_Urine_1561bp_2016 50
	75.22	76.61	77.11	74.78	77.38	74.3	74.78	77.35	74.84	75.33	(51)_Setif_Urine_1738bp_2016 51
	73.42	78.19	75.95	72.88	72.84	75.72	77.04	75.05	75.88	73.68	(52)_Setif_Urine_1215bp_2016 52
	70.27	76.55	76.53	73.48	76.94	76.04	79.9	67.85	76.04	76.73	(53)_Setif_Urine_1682bp_2016 53
	76.36	73.41	75.46	76.07	77.96	70.08	59.06	76.3	76.62	61.14	(54)_Setif_Urine_1561bp_2016 54
	69.76	75.14	77.69	75.37	77.83	76.49	78.86	70.56	77.83	78.96	(55)_BBA_Urine_1561bp_2016 55
	72.96	77.32	74.1	73.78	75.45	75.65	74.58	72.44	75	74.92	(56)_Setif_Urine_1680bp_2016 56
	71.88	77.13	77.79	72.78	76.87	78.8	77.9	69.62	76.11	75.58	(57)_Setif_Urine_1561bp_2016 57
	69.77	75.99	76.33	71.98	77.28	76.64	78.11	69.83	75.93	78.22	(58)_Setif_Urine_1558bp_2016 58
	75.66	73.86	77.79	75.77	59.05	77.98	77.53	73.9	57.12	77.31	(59)_Setif_Urine_1553bp_2016 59
	78.03	75.4	76.24	78.17	77.63	71.51	0.18	77.56	76.26	60.15	(60)_BBA_Urine_1693bp_2016 60
	76.56	77.78	77.4	77.47	71.53	75.26	79.34	75.05	71.35	76.04	(61)_Setif_Urine_1152bp_2016 61
	76.95	75.12	76.14	75.37	60.53	76.7	75.62	76.2	60.45	76.37	(62)_Setif_Urine_1207bp_2016 62
	76.18	73.04	75.85	75.97	77.81	70.26	71.56	76.93	78.05	72.28	(63)_Setif_Urine_1681bp_2016 63
	75.42	72.59	78.14	78.24	77.41	72.07	68.1	78.45	76.67	69.04	(64)_Setif_Urine_956bp_2016 64
	69.44	76.49	76.33	73.08	78	76.15	77.17	72.03	78.14	75.66	(65)_Setif_Urine_1656bp_2016 65
	0	75.78	74.1	72.78	75.33	77.25	78.03	69.52	76.72	75.33	(66)_Setif_Urine_1675bp_2016 66
		0	76.04	77.77	76.17	74.06	75.4	74.01	74.89	75.25	(67)_Setif_Urine_1565bp_2016 67
			0	46.46	78.37	74.59	76.04	74.63	78.18	76.14	(68)_Setif_Urine_1031bp_2016 68
				0	76.47	75.87	78.17	73.8	77.07	75.07	(69)_Setif_Urine_1003bp_2016 69
					0	79.55	77.5	77.14	59.35	76.98	(70)_Setif_Urine_1609bp_2016 70
						0	71.51	77.04	80	71.12	(71)_Setif_Urine_1685bp_2016 71
							0	77.56	76.14	60.31	(72)_Setif_Urine_1685bp_2016 72
								0	75.89	77.77	(73)_Setif_Urine_958bp_2016 73
									0	76.9	(74)_Setif_Urine_1685bp_2016 74
										0	(75)_Setif_Urine_1212bp_2016 75

Supplementary Figure 1 Multiple sequence alignment reveals a highly conserved N-terminal region (a) of the *fliC* sequences, significant variability in the central region (b), and a highly conserved C-terminal region (c). There are only five positions (*) that show strong identity within the sequences

Supplementary Figure 2 Multiple correspondence analysis for the pathogen *E. coli* from the three sampling provinces of Setif, Batna and Bordj Bou Arreridj
