# Supplementary figures and images for: Bioinformatics Analysis and Spatiotemporal Distribution of the fliC Gene and Its Protein Isolated from Escherichia coli-Infected Patients in Eastern Algeria
Source: Malays J Med Sci. 2024 Oct 8;31(5):161–95. doi: 10.21315/mjms2024.31.5.12 (PMC11477471; doi:10.21315/mjms2024.31.5.12)

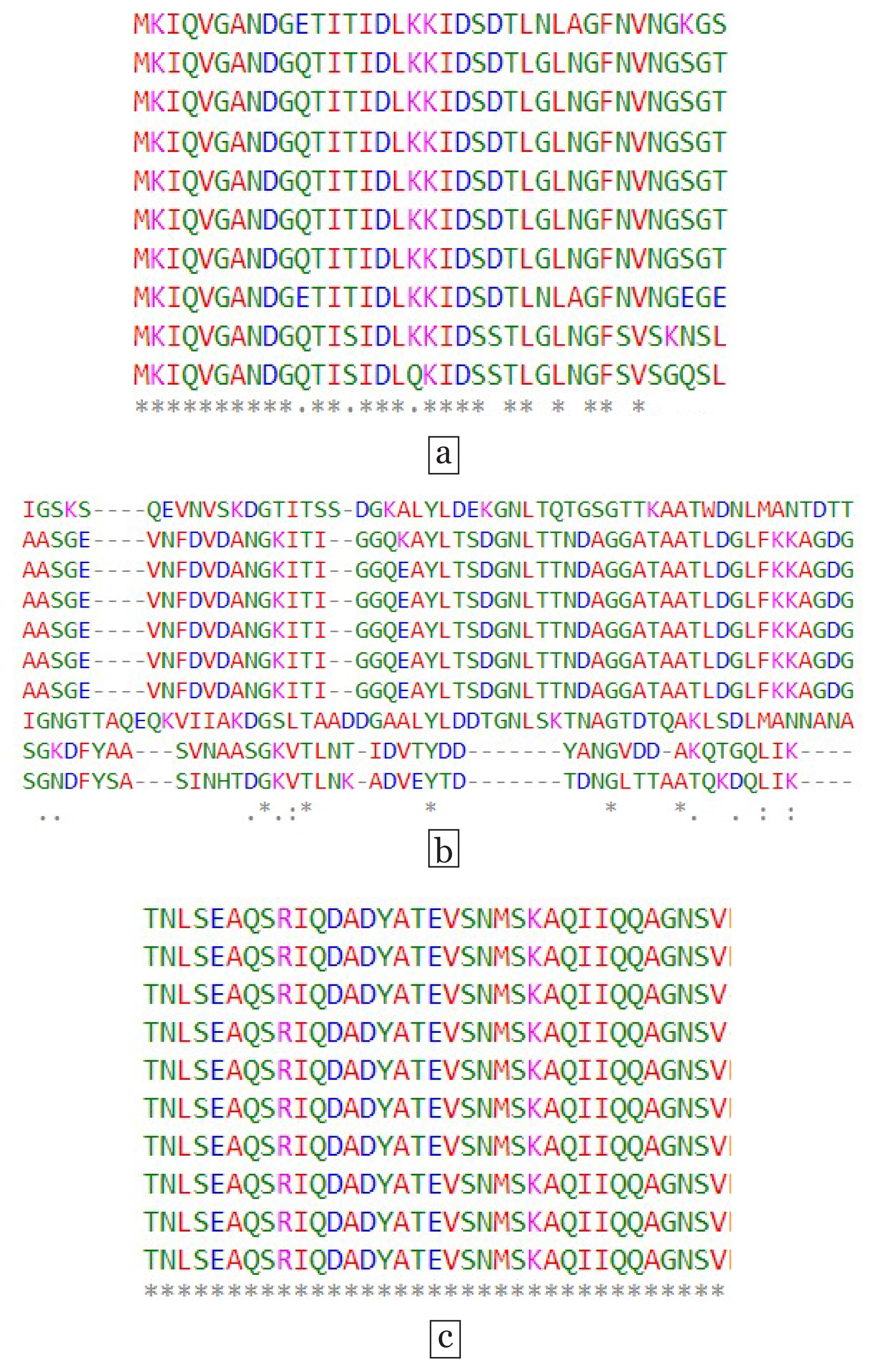

Supplement: Supplementary Figure 1 — Multiple sequence alignment reveals a highly conserved N-terminal region (a) of the fliC sequences, significant variability in the central region (b), and a highly conserved C-terminal region (c). There are only five positions (*) that show strong identity within the sequences [file 12mjms3105_oas1.tif]

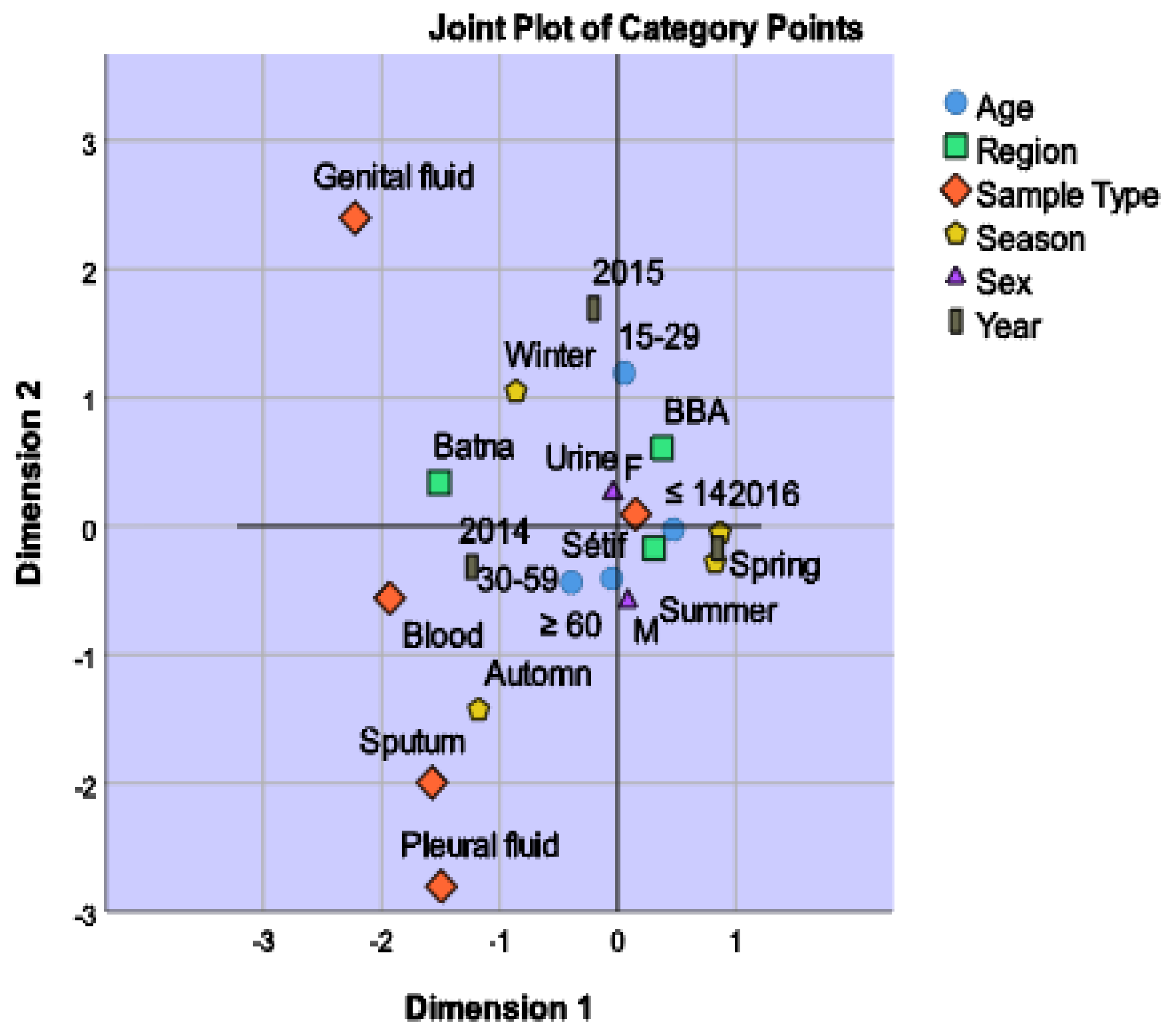

Supplement: Supplementary Figure 2 — Multiple correspondence analysis for the pathogen E. coli from the three sampling provinces of Setif, Batna and Bordj Bou Arreridj [file 12mjms3105_oas2.tif]
